# Vorsicht: bissig! Hundebissverletzungen in der klinischen Rechtsmedizin

**DOI:** 10.1007/s00104-022-01741-5

**Published:** 2022-10-25

**Authors:** V. Kolbe, R. Bingert, S. Märzheuser, A. Büttner

**Affiliations:** 1 Institut für Rechtsmedizin, Universitätsmedizin Rostock, St.-Georg-Str. 108, 18055 Rostock, Deutschland; 2 Klinik für Kinderchirurgie der Chirurgischen Klinik und Poliklinik, Universitätsmedizin Rostock, Rostock, Deutschland

**Keywords:** Klinische Rechtsmedizin, Befunddokumentation, Hundebissverletzungen, Wundverschluss, Prävention, Clinical forensic medicine, Documentation of findings, Dog bite injuries, Wound closure, Prevention

## Abstract

Insbesondere jüngere Kinder sind aufgrund ihrer geringen Körpergröße gefährdet, Opfer von Hundeangriffen zu werden. Ein solcher Beißvorfall kann verschiedene Strafbestände erfüllen. Um möglichen rechtlichen Ansprüchen genügen zu können, sollten die Verletzungen rechtsmedizinisch und damit gerichtfest dokumentiert werden. Es empfiehlt sich daher eine enge Zusammenarbeit von behandelnden ChirurgInnen und RechtsmedizinerInnen. Es wurde eine retrospektive Analyse der klinisch-rechtsmedizinisch bearbeiteten Fälle von Hundebissverletzungen bei Kindern und Erwachsenen an der Universitätsmedizin Rostock unter verschiedenen Aspekten durchgeführt. Erwachsene Verletzte wurden überwiegend an der unteren Extremität verletzt. Die untersuchten Kinder wurden überwiegend die Kopf‑, Hals- und Gesichtsregion sowie die obere Extremität gebissen. Die Verletzungsschwere reichte von Hautrötungen bis zu schweren Substanzdefekten mit Verlust von Körperstrukturen. Der beißende Hund war in gut der Hälfte der Fälle bekannt. Um Beißangriffe durch Hunde auf Kinder zu vermeiden, sollten Eltern sowie auch Kinder unbedingt im Umgang mit Hunden geschult werden.

## Abstract (en)

Younger children in particular are at risk of becoming victims of dog attacks due to their small size. Such a biting incident can fulfil various criminal offences. In order to be able to satisfy possible legal claims, the injuries should be documented by forensic medicine and thus be legally binding. Therefore, close cooperation between the treating surgeons and forensic experts is recommended. A retrospective analysis of the cases of dog bite injuries in children and adults treated by clinical forensic medicine at the University Medical Center Rostock was carried out under various aspects. Adult casualties were predominantly injured on the lower extremities. The children examined were predominantly bitten on the head, neck and face region as well as the upper extremities. Injury severity ranged from skin redness to severe substance defects with loss of body structures. The biting dog was known in about half of the cases. In order to avoid dog bites in children, parents as well as children should be trained in dealing with dogs.

## Hintergrund

In deutschen Haushalten lebten im Jahr 2021 etwa 34 Mio. Haustiere, darunter 12,27 Mio. Hunde. Sie gehören damit bundesweit zu den zweitbeliebtesten Haustieren nach Katzen [18]. Der Hund ist häufig nicht nur Haustier, sondern spielt als Weggefährte oder Familienmitglied eine wichtige Rolle. Deutsche Schäferhunde, Dackel und Deutsch Drahthaar zählten zu den beliebtesten Hunderassen, gefolgt von Labrador Retrievern, Pudeln und Golden Retrievern [33]. Bei einer so großen Hundepopulation verwundert es nicht, dass Verletzungen durch Hunde häufig auftreten. Ein Beißvorfall kann dabei verschiedene Rechtsgüter verletzen und unterschiedliche Strafbestände erfüllen. In Einzelfällen kann ein Hund als gefährlicher Gegenstand oder Waffe betrachtet werden [6]. Eine bundeseinheitliche „Beißstatistik“ existiert nicht, da es keine Meldepflicht gibt [25]. Eine Bissverletzung führt nicht zwangsläufig zu einer medizinischen Vorstellung, sodass genaue epidemiologische Angaben nicht zu erfassen sind. Die Häufigkeit tödlicher Hundebissverletzungen wird mit 3,3 Personen/Jahr beziffert [22].

Ein Beißvorfall kann neben dem Strafrecht für weitere Rechtsgebiete wie Verwaltungsrecht, Schadensrecht oder Ordnungswidrigkeitenrecht relevant sein. Die Verletzten, insbesondere Kinder, sollten daher rechtsmedizinisch begutachtet und die Befunde gerichtsfest dokumentiert werden. Dabei geht es weniger um eine Überprüfung des angegebenen Sachverhaltes, sondern mehr um die Beurteilung der Verletzungsschwere und -dynamik sowie der medizinischen Prognose.

Jüngere Kinder sind aufgrund ihrer geringeren Körpergröße besonders gefährdet, Opfer von Hundeangriffen zu werden [5, 7]. Sie sind unerfahren darin, Zeichen des Hundes korrekt zu deuten: Sie können nicht unterscheiden, ob ein Hund ängstliches, aggressives oder doch verspieltes Verhalten zeigt. Wegen ihrer geringen Körpergröße können sie in das Beuteschema von Hunden passen [7]. Größere, beißkräftige Hunderassen, wie Schäferhunde, Rottweiler oder Retriever – die regelmäßig die Beißstatistiken anführen –, verursachen überwiegend schwere Verletzungen an Kopf, Nacken und Hals von Kindern im Vorschulalter [3, 7, 23, 24, 27, 32, 36, 37]. Ältere und größere Kinder werden eher in Arme, Hände und Beine gebissen [11, 30].

Hundebissverletzungen können zu lebensgefährlichen Verletzungen führen [1, 3, 7, 8, 32, 36].

Die Bearbeitung und Behandlung von Hundebissverletzungen sind im (kinder-)chirurgischen Alltag häufiger als im rechtsmedizinischen. Die Bandbreite an Verletzungen reicht von oberflächlichen Quetsch-Riss-Wunden, Kratz- oder Schürfdefekten bis zu Abrissverletzungen und/oder großflächigen Substanzdefekten [3, 20]. Das Infektionsrisiko ist abhängig von der Art der Verletzung und der individuellen Infektionsdisposition des/der Gebissenen: Tiefe, verschmutzte Wunden mit großflächiger Gewebszerstörung, Verletzungen im Gesicht und/oder an den Extremitäten sowie Gewebe mit reduzierter Durchblutung bergen ein hohes Risiko, sich zu infizieren [28, 30]. Das Risiko einer Wundinfektion nach Hundebissverletzungen wird je nach Quelle mit 3–18 % bzw. 5–25 % [25, 38], nach einem sorgfältigen Wunddébridement mit etwa 2 % beschrieben [21].

Um ein kosmetisch zufriedenstellendes Ergebnis zu erzielen, sollten die Verletzungen nach gründlicher Reinigung mehrschichtig vernäht und antibiotisch flankiert werden [14, 34].

Im Folgenden sollen die rechtsmedizinisch bearbeiteten Fälle von Hundebissverletzungen bei Kindern und Erwachsenen an der Universitätsmedizin Rostock analysiert und mögliche Präventionsmaßnahmen abgeleitet werden.

## Material und Methoden

In die vorliegende Studie wurden alle nichttödlichen Fälle von Hundebissverletzungen und alle nicht näher bezeichneten Verletzungen durch Hunde eingeschlossen, die zwischen 2004 und 2021 am Institut für Rechtsmedizin der Universitätsmedizin Rostock bearbeitet wurden. Die erfassten Variablen der Geschädigten umfassten Alter, Geschlecht und vorbestehende Erkrankungen. Es wurden die verletzten Körperregionen, die Verletzungsschwere, die notwendigen medizinischen Maßnahmen und die Dauer des stationären Aufenthaltes analysiert. Es wurden die Rasse des bezichtigten Hundes, das Verhältnis der Verletzten zu dem Hund und die Beißumstände erfasst. Alle Behandlungsunterlagen wurden, sofern vorliegend, ausgewertet.

Statistische Analysen und Abbildungen wurden mittels Microsoft Excel (Version 2019) und GraphPad Prism (Version 8.0.1) für Windows vorgenommen.

## Resultate

Es konnten 19 Fälle anhand der beschriebenen Einschlusskriterien ausgewertet werden. Es fanden sich 17 Verletzte, die im Rahmen der niedrigschwelligen Gewaltopferambulanz untersucht wurden. Darunter waren zehn konsiliarische Aufträge verschiedener Kliniken der Universitätsmedizin Rostock, insbesondere der Kinder- und Jugendklinik und der Mund-Kiefer-Gesichtschirurgie sowie sieben private Aufträge durch die verletzten Personen selbst. Zwei körperliche Untersuchungen fanden im Auftrag der Ermittlungsbehörden statt. Eine rechtsmedizinische Begutachtung der Betroffenen erfolgte durchschnittlich 32 h nach dem Vorfall, wobei sich die Zeitspanne zwischen Untersuchung und Vorfallszeitpunkt von 2,5 h bis 4 Tage erstreckte.

Sieben der zu Untersuchenden waren männlich, zwölf Betroffene weiblich. Unter den insgesamt sieben betroffenen Kindern befanden sich sechs Mädchen und ein Junge.

**Figure 1:**
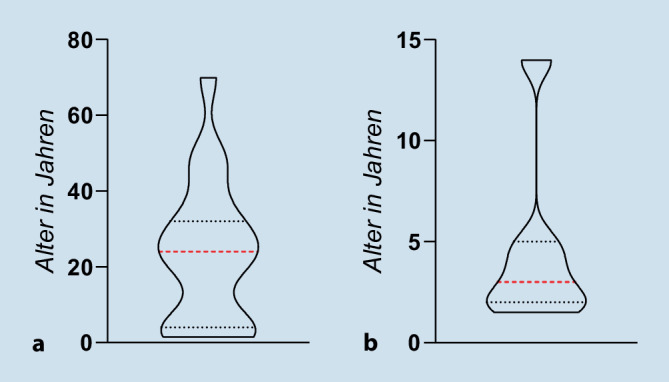

Wie Abb. 1 verdeutlicht, lag das mediane Alter bei 24 Jahren, wobei die Altersspanne von 1,5 bis 70 Jahren reichte. Die betroffenen Kinder waren im Median 3 Jahre alt (Minimum 1,5 Jahre, Maximum 14 Jahre).

### Beißsituationen und Umgebungsfaktoren

In zehn Fällen ereigneten sich die Beißvorfälle durch einen dem Betroffenen bekannten Hund, der entweder zum eigenen Haushalt gehörte bzw. im Besitz von Angehörigen oder Freunden war. In acht Fällen wurden die Verletzten durch fremde Hunde gebissen, denen sie zuvor nicht begegnet sind. In einem Fall konnten keine Daten zu der Beziehung von verletzter Person und Hund erhoben werden.

In drei Fällen wurden bereits in der Vergangenheit Beißvorfälle durch den gleichen Hund bekannt.

Von den betroffenen Kindern wurden sechs von einem bekannten Hund, darunter in zwei Fällen von dem Familienhund, angegriffen. Ein Kind wurde durch einen fremden Hund verletzt.

In mehr als der Hälfte der Fälle (52 %) ereignete sich die Beißsituation im privaten Umfeld. In 42 % der Fälle kam es zu einem Beißvorfall in der Öffentlichkeit. Zwei untersuchte Erwachsene waren selbst HundebesitzerInnen, die bei einem Spaziergang mit dem eigenen Tier von einem anderen Hund gebissen wurden. In einem Fall waren die näheren Beißumstände nicht bekannt.

Zwei der Bissopfer wurden während ihrer Tätigkeit als Paketzusteller – beim Betreten der Grundstücke der Sendungsempfänger – angegriffen.

Während der Beißvorfälle waren 47 % der Hunde nicht angeleint, 11 % trugen eine Leine. In 42 % der Fälle lagen keine Informationen bezüglich der Leinensituation vor.

Die Beißvorfälle ereigneten sich im Frühling (*n* = 8), gefolgt von den Sommer- (*n* = 5) und Herbstmonaten (*n* = 6). In den Wintermonaten ereignete sich keiner der erfassten Hundebisse.

### Verletzungsart, -verteilung und -schwere

Die Verletzungsanzahl lag im Mittel bei 3,4 mit einer Standardabweichung von 3,1. Dabei reichte die Spannweite von einer bis mehr als zehn Einzelverletzungen.

**Figure 2:**
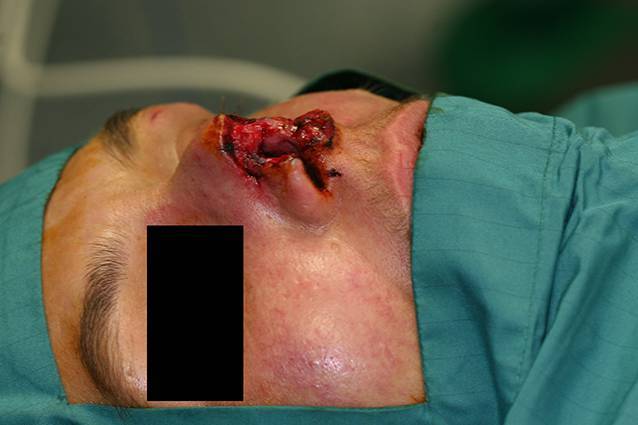

Bei erwachsenen Betroffenen wurde am häufigsten die untere Extremität verletzt (44 %). Bei 24 % der Erwachsenen fanden sich Verletzungen der oberen Extremität. In jeweils einem Fall konnten Verletzungen der Rumpfrückseite, des Gesäßes und des Genitals festgestellt werden. Mit 20 % waren Verletzungen im Kopf‑, Hals- und Gesichtsbereich seltener festzustellen als Verletzungen der oberen Extremität (Abb. 2).

Bei kindlichen Bissopfern befanden sich 57 % der Verletzungen im Bereich der Kopf‑, Hals- und Gesichtsregion und 42,8 % im Bereich der oberen Extremität. In 28,5 % der Fälle betroffener Kinder war die untere Extremität betroffen.

Unter Berücksichtigung der Klassifikation von Hundebissverletzungen des Gesichts bzw. Kopfes bei Kindern nach Lackmann [13] wiesen zwei Bissopfer oberflächliche Verletzungen entsprechend einem Stadium I auf (Tab. 1). Bei einem Kind lagen ausgeprägte Ohrknorpeldefekte mit Teilamputation im Sinne eines Stadium III vor. In einem Fall zeigten sich ausgedehnte Bissverletzungen, welche teilweise Nervenverletzungen – entsprechend einem Stadium IVa – zur Folge hatten.

**Table 1:** 

Fall	Alter (Jahre)	Geschlecht	Stadieneinteilung nach Lackmann [19]
4	3	♀	III
11	2	♂	I
12	1,5	♀	IVa
16	4	♀	I

**Figure 3:**
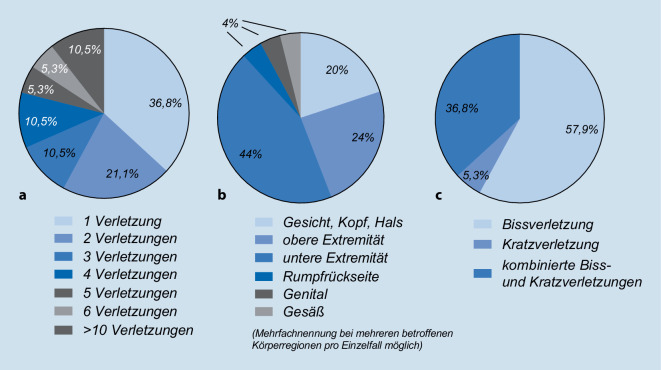

Bei 57,9 % aller Verletzten konnten isolierte Bissverletzungen begutachtet werden, wohingegen in 36,8 % der Fälle kombinierte Biss- und Kratzverletzungen und in einem Fall ausschließlich Kratzverletzungen vorlagen (Abb. 3 verdeutlicht).

In 84,3 % war ein Hund, in 15,7 % der Fälle waren zwei Hunde in die Beißvorfälle verwickelt. Hierbei zeigten sich in zwei Fällen zwei betroffene Körperregionen und in einem Fall besonders ausgedehnte Verletzungen (mehr als zehn Einzelverletzungen).

### Verletzungsschwere in Bezug auf die Hunderasse – Einzelfallbetrachtungen

In zehn der untersuchten Fälle wurden die Rassen der verursachenden Hunde bekannt. Eine Übersicht gibt Tab. 2.

**Table 2:** 

	Schäferhund	Rottweiler	Doggenmischling	Border-Collie	Alternative Bulldogge	Pitbull	Bullmastiff	Australien Shepherd-Mischling	Shi Tzu
Alter der Person (Jahre)	24	14	48	2	1,5	21	32; 3	4	2
Anzahl der Vorfälle	1	2	2	2	1	1	2	1	1
Bevorzugte Verletzungsregion	Untere Extremität	Obere Extremität	Untere Extremität	Gesicht	Gesicht	Untere Extremität	Gesicht, obere Extremität	Gesicht	Obere Extremität
Verletzungsintensität	Gering	Mittelgradig	Gering- bis mittelgradig	Mittelgradig	Hochgradig lebensbedrohlich	Gering	Hochgradig	Mittelgradig	Gering

Im Folgenden sollen diese Fälle kurz skizziert werden. Die Angaben zu den Vorfällen beruhen vorwiegend auf den Angaben der Geschädigten bzw. deren Angehörigen.

#### Fall A.

**Figure 4:**
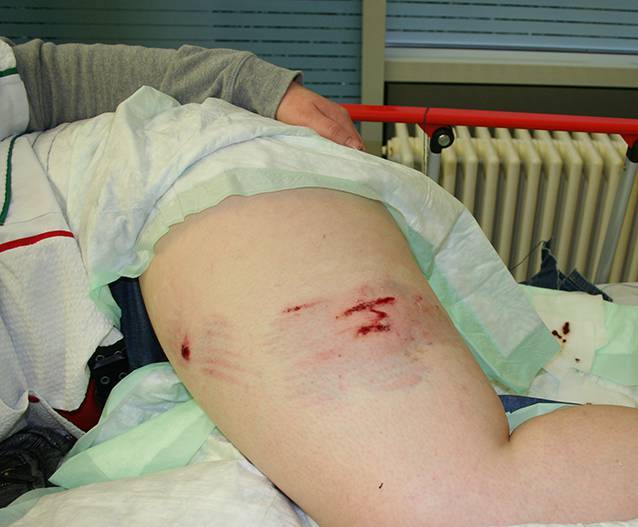

Eine 24-jährige Frau sei in den Morgenstunden auf dem Grundstück ihres Partners auf dem Weg zu ihrem KFZ gewesen, als sie von dem **Schäferhund** der Familie ins Bein gebissen worden sei. Der Cousin des Freundes habe die Tiere auf dem Grundstück versorgen wollen und hätte den ihn begleitenden Hund dabei nicht angeleint. Der Hund habe die Frau am Bein „festgehalten“ und sich nicht durch den Halter abrufen lassen. Sie erlitt dadurch oberflächliche bisstypische Schürfungen und Kratzverletzungen am Oberschenkel (Abb. 4).

#### Fall B.

Ein 14-jähriges Mädchen habe einen Freund besucht und sei durch dessen **Rottweiler** ohne erkennbaren Grund in den Oberarm gebissen worden. Sie erlitt dadurch penetrierende Bisswunden am beugeseitigen sowie streckseitigen Oberarm, außerdem zahlreichen Kratzverletzungen am Oberarm und dem achselnahen Brustkorb. Das Mädchen sei eine Woche zuvor durch denselben Hund verletzt worden.

#### Fall C.

Eine 48-jährige Hundebesitzerin sei mit ihrem Hund auf einem Feld spazieren gewesen, als sie auf ein Paar mit zwei Hunden getroffen sei. Alle drei Hunde seien nicht angeleint gewesen und hätten zunächst miteinander gespielt, bis der Mann zwischen die Tiere gegangen sei, seine Frau habe geschrien. Die später Betroffene habe ihren Hund daraufhin angeleint. Dabei sei sie von einem **Doggenmischling** 2‑mal ins Bein gebissen worden. Ihr Hund sei auch verletzt worden. Die Frau erlitt zackenförmige, tiefe Bissverletzungen am medialen und lateralen Unterschenkel.

#### Fall D.

Ein 2‑jähriger Junge habe sich mit seinem Großvater auf dem Grundstück befunden und am Zaun einen Bagger beobachtet, als der **Border Collie** der Kindseltern das Kind ins Gesicht gebissen habe. Der Großvater habe den Hund sofort weggetreten. Der Junge erlitt eine klaffende Bissverletzung am Mundwinkel und der Wange sowie sturztypische Verletzungen an der Stirn. Der Hund sei abgegeben worden.

#### Fall E.

Ein 1,5 Jahre altes Mädchen sei durch nicht bekannte Umstände durch den Familienhund, eine **Alternative Bulldogge**, angegriffen worden. Das Kind erlitt dabei schwere Bissverletzungen hemifazial, zervikal, nuchal und femoral. Wundinfektionen der Wange und des Oberschenkels mit *Pseudomonas aeruginosa* machten Wundrevisionen erforderlich. Der Hund sei noch vor Ort eingeschläfert worden.

#### Fall F.

Ein 21-jähriger Asylbewerber sei nachts auf mehrere Personen getroffen, die ausländerfeindliche Parolen gerufen hätten. Es sei zu einer Auseinandersetzung gekommen, bei der der junge Mann mit einem Schlagring sowie einem Kettenschloss verletzt worden sei. Einer der Angreifer habe seinem **Pitbull** etwas zugerufen, woraufhin dieser den jungen Mann ins Bein gebissen habe. Neben den Verletzungen durch die beschriebenen Waffen erlitt der Betroffene oberflächliche Schürfungen am Unterschenkel.

#### Fall G und H.

**Figure 5:**
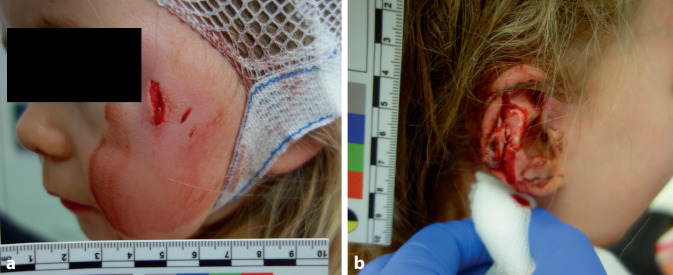

Ein 3‑jähriges Mädchen habe im Garten der Familie gespielt, als es von einem **Bullmastiff** angegriffen worden sei, der sich zuvor auf Höhe des Grundstückes von der Leine gerissen habe und zielgerichtet auf das Kind zugelaufen sei. Das Kind erlitt Bissverletzungen infraorbital rechts und lateroorbital links sowie eine Teilamputation des Ohres (Abb. 5). Das Tier sei durch die 32-jährige Kindsmutter sowie einen Nachbarn unter Kontrolle gebracht worden. Die Mutter erlitt dabei Kratzverletzungen an den Armen, Händen und den Beinen.

#### Fall I.

Ein 4‑jähriges Mädchen habe mit ihrer Großmutter deren Schwester besucht. Als man sich im engen Flur anziehen wollte, habe das Kind den **Australien Sheperd-Mischling** der Großtante streicheln wollen. Der Hund sei aufgrund des bevorstehenden Spazierganges aufgeregt gewesen. Der Rüde habe das Mädchen umgerannt und ihr in das Gesicht gebissen. Sie erlitt Bissverletzungen an der Wange.

#### Fall J.

Ein 2‑jähriges Mädchen habe im Haushalt der Urgroßmutter den nicht kindgewöhnten **Shi Tzu** der Großtante streicheln wollen, der sich unter einem Stuhl versteckt habe. Das Mädchen sei zu dem Hund unter den Stuhl gekrochen und dabei in die Hand gebissen worden. Sie erlitt hierbei Rötungen an den Fingern.

### Medizinische Versorgung

Von den acht stationär behandelten verletzten Personen wurden sechs in der Universitätsmedizin Rostock und zwei in auswärtigen Kliniken versorgt. In fünf Fällen erfolgte eine ambulante medizinische Behandlung der Verletzungen. In einem Fall wurde der Verletze nur ambulant in einem Rettungswagen versorgt, in vier Fällen fand keine klinische Vorstellung statt.

In 15 Fällen konnten medizinische Behandlungsunterlagen mitbeurteilt werden.

Eine Übersicht über die erforderlichen medizinischen Maßnahmen gibt Tab. 3.

**Table 3:** 

Fall	Alter (Jahre)	Geschlecht	Stationäre Aufnahme erfolgt	Dauer	Antibiose erfolgt	Naht erforderlich	Weitere Therapie	Besonderheiten
1	24	♀	Nein	–	–	Nein	–	–
2	24	♀	Nein	–	k. A.	Nein	–	–
3	32	♀	Nein	–	Nein	Nein	–	–
4	3	♀	Ja	–	Ja	Ja	Plastische Rekonstruktion	–
5	14	♀	Nein	–	Ja	Ja	Wundspülung, Anlage Gilchristverband	Ambulante Behandlung
6	70	♂	Ja	2 Tage	Ja	Nein	Desinfektion, Salbenverbände	–
7	48	♀	Nein	–	Ja	Nein	Röntgen, Wundspülung	–
8	30	♂	Nein	–	Ja	Nein	Analgesie, Tetanusimpfung	D‑Arzt-Vorstellung
9	49	♀	Nein	–	Ja	Nein	Wundspülung, Wundverband mit Zweifingergipsschiene	Ambulante Behandlung
10	5	♀	Nein	–	Nein	Nein	Wunddesinfektion, steriler Verband	Ambulante Behandlung
11	2	♂	Ja	4 Tage	Ja	Ja	–	–
12	1,5	♀	Ja	17 Tage	Ja	Ja	Primäre Wundversorgung mit Rekonstruktion und kombinierter Lappenplastik	Wundinfektion (multiresistenter *Pseudomonas aeruginosa*)
13	32	♂	Nein	–	Ja	Nein	Wundspülung, leichte Wundadaption	D‑Arzt-Vorstellung, ambulante Behandlung
14	21	♂	Ja	k. A.	k. A.	Nein	–	Medizinische Behandlung aufgrund anderer Verletzungen erforderlich
15	22	♂	Ja	k. A.	k. A.	Nein	–	Medizinische Behandlung aufgrund anderer Verletzungen erforderlich
16	4	♀	Ja	–	Ja	Ja	–	–
17	25	♂	Nein	–	Nein	Nein	–	Medizinische Behandlung lediglich im RTW erfolgt, Polizei vor Ort
18	40	♀	Ja	7 Tage	Ja	Ja	Plastische Rekonstruktion erforderlich Thromboseprophylaxe	–
19	2	♀	Nein	–	Nein	Nein	–	–

## Diskussion

Tierbissverletzungen gehören zu den vermeidbaren traumatischen Verletzungen. Übereinstimmend mit internationalen Studien konnte auch in der vorliegenden Untersuchung gezeigt werden, dass große Hunderassen deutlich häufiger in Beißvorfälle verwickelt waren, wenngleich die Rasse nicht in allen Fällen benannt werden konnte [10, 27, 37]. In der überwiegenden Zahl der Fälle handelte es sich um den Familienhund bzw. um einen der verletzten Person bekannten Hund, was sich mit den Ergebnissen anderer Studien deckt [3, 11]. Die vergleichsweise geringe Fallzahl steht in deutlichem Kontrast zu anderen internationalen Studien [9], erklärt sich aber auch durch die geringe Zahl an wilden bzw. streunenden Hunden in Deutschland.

Neben ihrer geringen Körpergröße, mit der sie in ihr grobes Beuteschema passen, können unkoordinierte Bewegungsreize und laute Geräusche von Kindern plötzliche, unerwartete Angriffe von Hunden auslösen. Gleichzeitig können kleine Kinder das Verhalten, insbesondere körperliche und akustische Drohgebärden von Hunden nicht oder nicht korrekt einschätzen und die Tiere unbewusst bedrängen, sodass zahlreiche Beißvorfälle nur vermeintlich „unprovoziert“ waren [6, 7]. Neben rassetypischen Charakterzügen kann eine gesteigerte Aggressivität durch die Lebensumstände sowie die entsprechende Erziehung des Hundes gefördert oder kontrolliert werden.

Dabei können unterschiedliche Aggressionsformen unterschieden werden: Eifersucht oder Dominanzverhalten, z. B. gegenüber neuen Familienmitgliedern, beschützendes Verhalten bei vermeintlicher Gefahr für das „Rudel“, aber auch bei der Wegnahme von Futter oder Spielzeug.

Territoriales Verhalten des Hundes kann ebenfalls zu einem gesteigerten Aggressionspotenzial führen [3]. PostbotInnen oder PaketzustellerInnen sind besonders gefährdet, Opfer von Beißvorfällen zu werden, wenngleich keine offizielle Statistik zur Häufigkeit existiert. Auch in unserem Untersuchungsgut fanden sich zwei bei der Ausübung dieses Berufes Verletzte. Durch das zielstrebige Eindringen in ihr „Revier“ können Hunde verunsichert oder verängstigt werden und aggressiv reagieren. Kommt es im Rahmen der Zustellertätigkeit zu einem Hundebiss, liegt ein Arbeitsunfall vor. In solchen Vorfällen haften die HundehalterInnen verschuldensunabhängig (§ 833 Bürgerliches Gesetzbuch (BGB)).

Misshandelte Hunde, die Angst oder Schmerzen haben, können ebenfalls aggressiv reagieren. In einigen Fällen bleibt der Auslöser ungeklärt. In diesen Fällen sollte an genetische, infektiöse oder neurologische Erkrankungen gedacht werden sowie toxikologische Beeinflussungen ausgeschlossen werden [3].

Tödlich verlaufende Beißvorfälle sind im Untersuchungszeitraum in den Landgerichtsbezirken Rostock und Schwerin rechtsmedizinisch nicht bearbeitet worden. Dass Verletzte nach Hundebissverletzungen nach § 81 c StPO rechtsmedizinisch untersucht wurden, stellte die absolute Ausnahme dar. Der überwiegende Anteil der Betroffenen wurde im Rahmen klinischer Konsile oder auf eigenes Bestreben in der hiesigen Gewaltopferambulanz untersucht. Da rechtsmedizinische Ambulanzen eine gerichtsfeste Befunddokumentation für alle Geschädigten anbieten, dominieren in unserem Untersuchungskollektiv, wie auch in dem anderer rechtsmedizinischer Arbeiten [11], die Erwachsenen. Insgesamt waren in diesem Untersuchungskollektiv mehr Frauen und Mädchen Opfer von Beißvorfällen als Männer und Jungen. Bissverletzte Kinder wurden ausschließlich konsiliarisch mitbegutachtet. Dies entspricht auch den Ergebnissen anderer Studien, denen zufolge sich Bissopfer abhängig von der Schwere der Verletzungen medizinische Hilfe suchen, Kinder jedoch deutlich niedrigschwelliger medizinisch vorgestellt werden [11, 30]. Die Befunde der untersuchten Kinder und Jugendlichen fanden sich überwiegend an der oberen Körperhälfte – insbesondere am Kopf – und waren – übereinstimmend mit vergleichbaren Studien – schwerwiegender als die der untersuchten Erwachsenen [9, 15]. Erwachsene hatten einzelne Verletzungen an der unteren Körperhälfte, die geringfügige medizinische Versorgung benötigten. Dies entspricht auch den Feststellungen internationaler Studien [8, 9, 20]. Es darf davon ausgegangen werden, dass minderschwere Verletzungen – insbesondere von Familienangehörigen – nicht fachärztlich versorgt und bekannt gemacht werden, um mögliche behördlichen Konsequenzen zu vermeiden. Darin mag auch begründet sein, dass im vorliegenden Kollektiv vergleichsweise selten und nur bei schwerwiegenden Verletzungen der Familienhund verantwortlich gemacht wurde.

Im ländlich geprägten Mecklenburg-Vorpommern lebten 2021 220.000 Hunde [19]. Die geringe Fallzahl von Hundebissverletzungen im rechtsmedizinischen Kollektiv ist vor diesem Hintergrund nicht nachvollziehbar. Bei dieser großen Anzahl von Tieren muss von einer deutlich höheren Dunkelziffer ausgegangen werden, in denen Hunde ihre Halter oder andere ihnen bekannte Personen angegriffen und verletzt haben, ohne dass die Beißattacke angezeigt wurde.

**Figure 6:**
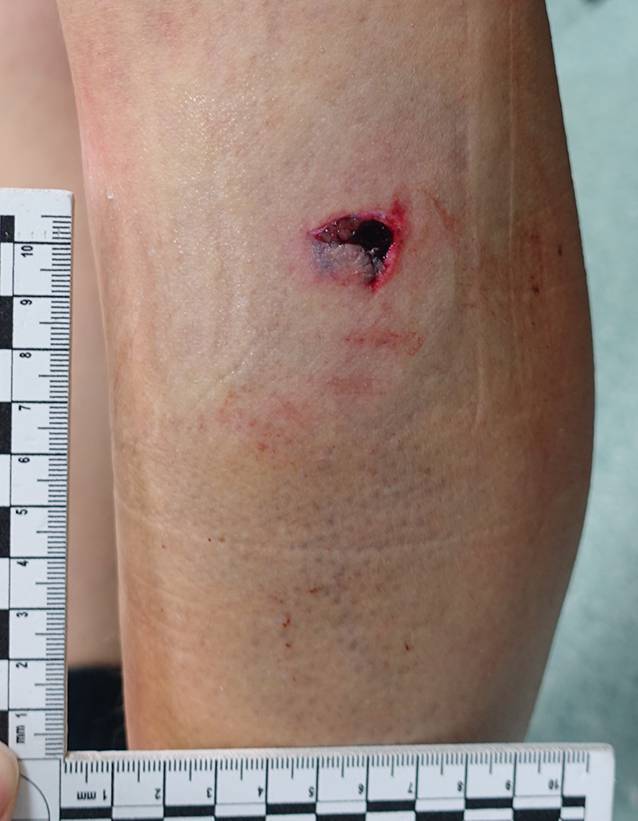

Hundebissverletzungen sind in ihrem Ausmaß äußerst variabel und können von leichten, oberflächlichen Hautdefekten über massive Verletzungen bis zum Tod führen [1, 7, 12]. Durch die unterschiedliche Stellung der Kiefer zueinander, die sich bei lang-, kurz- und rundköpfigen Hunderassen unterscheiden, ergeben sich unterschiedliche theoretische Beißkräfte [12]. Die langen Eckzähne der Tiere können punktförmige, penetrierende Gewebsdefekte verursachen, die durch die benachbarten Zähne von umgebenden Wundhöhlen begleitet werden, wie z. B. in Abb. 6 dargestellt. Kinder können aufgrund ihrer geringen Körpermasse von den angreifenden Hunden gepackt und geschüttelt werden, was zu massiven Gewebszerreißungen oder -ausrissen sowie Knochenbrüchen führen kann. Neben den Bissverletzungen, Gewebsquetschungen und -schürfungen durch Scherbewegungen können parallel verlaufende Kratzdefekte durch die Krallen der Hunde festgestellt werden [1, 3, 7, 15]. Ausgedehnte Verletzungen können zu Narben abheilen, die zu einer lebenslangen ästhetischen und funktionellen Beeinträchtigung bei den Betroffenen führen [28].

Das breite Spektrum an aeroben und anaeroben Keimen der beißenden Tiere stellt ein hohes Risiko für splenektomierte sowie immunsupprimierte Personen dar [37]. Derzeit gilt die Empfehlung, frische Bissverletzungen nach genauer chirurgischer Exploration primär zu verschließen und mit Amoxicillin und Klavulansäure zu therapieren. Außerdem sollte der Tetanusstatus überprüft und ggf. geimpft werden [26, 28, 34]. In unserem Kollektiv wurde ein primärer Wundverschluss bei sechs Personen durchgeführt und in elf Fällen antibiotisch flankiert.

Eine Hundehaltung erfordert eine nicht zu unterschätzende Übernahme von Verantwortung für den Hund, andere Personen und nicht zuletzt sich selbst. HundehalterInnen haften sowohl straf- als auch zivilrechtlich für Schäden, die der eigene Hund verursacht hat. Eine gefährliche Körperverletzung durch Hundebisse kann in Betracht kommen, wenn das Tier gemäß § 224 StGB als Waffe eingesetzt wird und auf andere Personen gehetzt wird. Derartige Sachverhalte wurden in vorliegendem Kollektiv in zwei Fällen von Verletzten beschrieben, die in behördlichem Auftrag untersucht wurden.

Es sind Risikofaktoren für Beißvorfälle bekannt, die sich auch in den ausgewerteten Fällen teilweise bestätigt haben: Neben der Hunderasse, Alter und Geschlecht des Tieres, dem Vorfallsort, dem Alter des Bissopfers und dessen Kenntnis über Hunde ist auch die Ausbildung des Hundes relevant [2]. Durch den Besuch einer Welpen- und/oder Junghundeschule kann die Beißhemmung gegenüber anderen Hunden und Menschen gefördert werden. Sozialisierte Tiere müssen einen größeren Widerstand überwinden, um auf einen Befehl hin zuzubeißen. Hunde, die schon einmal zugebissen haben, haben eine geringere Hemmschwelle. Ihre Bisse führen zu schwerwiegenderen Verletzungen als die von „Erstbeißern“ [6]. In der vorliegenden Erhebung waren in drei Fällen Beißvorfälle durch den gleichen Hund vorbeschrieben worden. In drei Fällen waren mehrere Hunde beteiligt. Der Jagdinstinkt wird im Rudel gefördert, die Beißfreudigkeit erhöht und die Hunde erschwert abrufbar [6]. Während die meisten Risikofaktoren nicht beeinflusst werden können, kann auf die Erziehung des Hundes maßgeblich eingewirkt werden.

Die Versicherungspflicht ist für HundehalterInnen in Deutschland nicht einheitlich geregelt, sondern obliegt den einzelnen Bundesländern. In Mecklenburg-Vorpommern existiert derzeit keine Versicherungspflicht. Unabhängig von juristischen Konsequenzen können Beißvorfälle zu einschneidenden Folgen für die HundehalterInnen führen: Über die zuständigen Veterinärämter können Auflagen nach den landesrechtlichen Hundegesetzen erteilt werden, die bis zur Wegnahme oder Euthanasie des beißenden Hundes führen können.

## Schlussfolgerungen

Verletzungen durch Hunde sind in Deutschland selten. Während der Corona-Pandemie nahm die Zahl an Hunden in deutschen Haushalten zu: Bei der Tierschutzorganisation Tasso e. V. wurden 2020 25 % mehr Hunde registriert als 2019 [35]. Gleichzeitig wurde die Ausbildung in zahlreichen Hundeschulen während der Lockdowns ausgesetzt. Es ist also zu befürchten, dass zahlreiche Hunde sowie ihre HalterInnen nur mangelhaft ausgebildet sind.

Wenngleich einzelne Rassen in den Beißstatistiken dominieren, kann grundsätzlich jeder Hund situationsabhängig zubeißen, sodass in der Aufarbeitung der Fälle die Gesamtsituation einschließlich der Haltungsbedingungen berücksichtigt werden sollte.

Wie Kienesberger et al. jüngst zeigen konnten, können Präventionsprogramme die Häufigkeit schwerer Verletzungen bei Grundschulkindern effektiv reduzieren [10], während das Gefahrenpotenzial vermeintlich harmloser Rassen tendenziell unterschätzt wird [17]. Es ist wünschenswert, dass Präventionsangebote pädagogischem Personal und Lehrkräften flächendeckend zur Verfügung gestellt werden. Eltern kleiner Kinder sollten geschult werden, um potenziell gefährliche Situationen korrekt einschätzen zu können. Die Deutsche Veterinärmedizinische Gesellschaft e. V. hat ein länderübergreifendes, computergestütztes Präventionsprojekt für Kinder zwischen 3 und 6 Jahren entwickelt, das sowohl ein Elternbegleitbuch als auch einen Leitfaden für ErzieherInnen und Lehrkräfte umfasst [4].

Wie aus Tab. 2 deutlich wird, werden kleinere Kinder überwiegend in das Gesicht bzw. die obere Extremität gebissen. Für die chirurgische Versorgung dieser Bissverletzungen gilt daher, dass eine sorgfältige Reinigung und ein penibler Wundverschluss für einen komplikationsfreien Verlauf und ein kosmetisch ansprechendes Ergebnis entscheidend sind [14, 28]. Nach Möglichkeit sollte – insbesondere bei Gesichtsverletzungen – ein mehrschichtiger primärer Wundverschluss unter flankierender Antibiose erfolgen [29], selbst wenn die klinische Vorstellung verzögert erfolgt [16, 31]. Bei Defektwunden sollten die fehlenden Körperteile gesucht und replantiert werden.

Nach der chirurgischen Versorgung der Verletzungen kann eine ergänzende rechtsmedizinische Befunddokumentation und -bewertung Verletzungen identifizieren, für die fälschlicherweise ein Hund verantwortlich gemacht werden soll, oder Biss- von Kratzverletzungen abgrenzen. Um Schmerzensgeldansprüche oder anderen Schadensersatz geltend machen zu können, muss der Biss nachgewiesen werden. Insofern empfiehlt es sich sowohl für die geschädigten Personen als auch die HundehalterInnen, zeitnah Beweise des Bisses – idealerweise rechtsmedizinisch – dokumentieren zu lassen, um auch von falschen Vorwürfen entlasten zu können. Da die rechtsmedizinische (Mit‑)Beurteilung meist verzögert erfolgt, sollten auch die Erstversorger die Verletzungen anhand der beschriebenen Charakteristika korrekt zuordnen und (foto‑)dokumentieren können [12].
